# Fighting for Equal Spiritual Voice: The Case of the “Women of the Wall”

**DOI:** 10.3389/fpsyg.2019.02199

**Published:** 2019-10-18

**Authors:** Shir Daphna-Tekoah, Rachel Sharaby

**Affiliations:** ^1^Ashkelon Academic College, Ashkelon, Israel; ^2^Kaplan Medical Center, Rehovot, Israel

**Keywords:** gender equality, religious feminism, Women of the Wall, Listening Guide, methodology, women’s rights

## Abstract

Our study contributes to the ongoing debate about women’s rights and religious feminism. The context for analysis of women’s experiences is the “Women of the Wall” who have been struggling for the past 30 years for their right to practice their spiritual rituals (praying at the Western Wall) in a hegemonic and masculine arena. We suggest that the “Women of the Wall” and their battle for spiritual equality threaten the hegemonic masculinity. Moreover, this feminist battle expands the feminist revolution and the fights for women’s equality to the religious arena which is dominated by hegemonic masculinity. The implementation of the Listening Guide, a feminist methodology, assists us in uncovering various voices, representing different aspects of the experiences of the “Women of the Wall” in a conflict zone. These narratives reveal juxtapositions of feelings of competence, determination and vulnerability and shed light on the women’s struggle for gender equality in a hegemonic masculine domain.

*“Any great change must expect opposition because it shakes the very foundation of privilege.” Mott, 2017*, *Suffrages*

## Women’s Struggles for Justice

There is a seemingly clear connection between the liberal democratic doctrine and the idea of feminism. The principle of the universal equality of human rights in the liberal democracy is compatible with the idea of feminism which can be defined as an idea that promotes the human rights and the civil rights of all women in the society as defined in 1978 within the framework of the “Declaration of the Rights of Man and of the Citizen” during the French Revolution and in 1948 in the “Universal Declaration of Human Rights” of the United Nations (Fogiel-Bijaoui, 2011). Liberal democracy also comprises the fertile soil which is essential for the feminist idea to develop and be influential.

However, a study of history leads to the conclusion that this connection was not clearly and simply actualized. The complexity of the relation between democracy and feminism is expressed in the fact that today, in the early 21st century, representation of women in centers of power is generally low. Furthermore, despite the democratization processes that characterized the late 20th century and early 21st century, the human rights of women in the world are still systematically and massively violated. The freedom of many women is still very limited, *inter alia* because they do not have the basic right of deciding on issues pertaining to their body and their sexuality. The rights of a large number of women in the world to property and possession is also very limited (Smith, 2000).

Feminist scholars brought to light important overlooked links between citizenship, rights, and gender. Feminist analyses try to expand and challenge the way in which scholars understand central concepts, such as power, security, and insecurity. They also bridge between theory and reality by refocusing our interest on other forms of power, such as women’s rights and women’s insecurities, into mainstream political science and gender studies (Harel-Shalev and Daphna-Tekoah, 2016). Åhäll (2012), Kronsell (2012), and others suggested that a specific methodology is needed for studying women in a male-dominated socio-political environment. Hansen (2006) proposed a discourse analytical framework and suggested that scholars be sensitive to competing identity-based discourses. Shepherd (2012) argued that scholars need to be attentive to the narrative through which individuals make sense of their experiences and the manner in which individuals understand their own realities. Gilligan (2015) suggested that scholars should be attentive to various hidden voices.

We followed these paths and aimed to extend these epistemological perspectives to unveil the Women of the Wall’s experiences and narratives, along with their voices, conflicts, and tonalities. We extended this focus to women who choose to participate in socio-political-religious activities for religious freedom in a hierarchic patriarchal environment.

Women’s struggles for justice influenced democratization processes in the world and were accompanied by developments that support women’s human and civil rights. One of the noteworthy developments is the trend of an increase in the average representation of women in centers of power and authority around the globe, which is expressed in ensuring representation for women (securing), electing women to senior roles and consolidation of the human rights of women as part of the political agenda (Eschle, 2001; Cook, 2012; Goetz and Jenkins, 2018).

The institution of citizenship that imparts membership in the national community comprises a foundation for belonging and a basis for solidarity between its members. As such, it creates inclusion and exclusion mechanisms in the community for different groups of the population, which are dynamic and given to change at the national, regional, and global level (Fogiel-Bijaoui, 2011).

Feminism as an idea that advances the human rights and the civil rights of all women in society also comprises a social movement. Feminism thus challenges the patriarchal discourse and the mechanisms that work for its institutionalization in various aspects. For example, combat is a masculine and patriarchic discourse and refers only to military combat, from which most women are excluded. The patriarchic discourse excludes women who fight against intimate violence, rape, and sexual harassment and does not refer to them as warriors, but rather as victims (Enloe, 2013; Daphna-Tekoah and Harel-Shalev, 2017; Harel-Shalev, 2017). Therfore, it is an action space in which organized activity of women who act within different frameworks takes place in the spirit of human rights in order to shape the social order to their advantage. In the political arena, this activity is intended to end men’s privileges and to include women in all of society’s institutions, including in the family. As such, feminism is opposed to any definition for a situation which is dictated by men for women.

In her feminist thinking, Simone de Beauvoir exposed the social mechanism that led to the subordination of women to men in the patriarchic discourse (De Beauvoir, 2001, 2007). She claimed that “man” and “woman” are not “natural” or biological traits, but are rather cultural categories, and that femininity and masculinity are products of education. She explained how biological differences between men and women were used over the course of history as justification for discrimination against women. In her opinion, there exists a fundamental contradiction in women’s attitude toward themselves. On the one hand, they aspire to express their personality and act in the world as free people. On the other hand, they accept the patterns and values of human (i.e., masculine) society that perceives the woman as an object. De Beauvoir also severely criticized the women themselves, who cooperate with the patriarchic system and educate their daughters into this suppressive system. The essential conditions which de Beauvior presented for the liberation of women are changing the laws and institutions, economic changes, and above all moral and cultural changes. Moreover, the role of the lesbian movements within the feminist movement was significant and influential. However, lesbian women are largely invisible and still suffer from discriminations (Salvati et al., 2018).

The feminists’ call to blur between the political-personal and the public-private-domestic is a call to undermine the fundamental assumption of the hierarchy that is perceived as modern and the world that is perceived as traditional (Herzog, 2009; Bunch, 2018; Levi-Hazan and Harel-Shalev, 2019). Feminist criticism has exposed the mechanisms that preserve patriarchy. In her viewpoint, there was no room for women as free people in the new public order in the modern age, in spite of declarations of equality and freedom. A new thinking scheme has established a liberal approach that defined an equation between the system’s needs and natural gender traits in universal and ahistoric terms. This equation became a powerful political mechanism for excluding women from the public sphere and reducing their contribution, appreciation and value in the social-public field.

The feminist discourse, similarly to other fields of discourse, is also an arena of struggle over boundaries and meaning. However, the logical thread that is intertwined between its different streams is use of knowledge which is located as source for analyzing social inequality and interrelations between the excluding and excluded forces (Hill-Collins, 2000). This analysis is made out of theoretical and empirical analyses in order to extract the human experience that was pushed to the margins and to use the knowledge that stems from experiences on the margins in order to re-examine the social orders that are constantly re-organized and mark different margins (Ellison, 1997; Love et al., 2019). The demand for different knowledge not only creates a different social reality, but also a different political reality. The reflexive aspect of feminist logic is the political position that demands constant exposure of the suppression and exclusion mechanisms of different groups, and constant turning to the margins and to the relations between marginal groups and central groups (Zwingel, 2016).

The construct feminism thus stresses women’s organized resistance to their subordination in any given society. It is no wonder that feminism has been one of the main actors in the public arena that is challenging the exclusion mechanisms established by the liberal civilian institution for over two decades (Eschle, 2001; Handrahan, 2004). Thus, as a philosophy or as a movement, feminism acts with the aim of advancing women’s human rights. An example for this is the feminist movement’s revolution in the 19th century to achieve the right to vote, after a prolonged and difficult struggle of suffragettes that even cost some women who insisted on equal rights in voting their lives. Today feminism is still challenging the liberal democratic citizenship with the goal of connecting between it and women’s human rights, in different forms. We are thus witness to different organizations of women who act to create a new religious discourse that connects to women’s human rights based on a multicultural version of liberal democracy (Razavi, 2000; Fogiel-Bijaoui, 2011; Enloe, 2013).

Similar processes in the civil and religious discourse are taking place in Israel. A change in women’s political civil status before the establishment of the State in 1948 (the first wave of Israeli feminism) enabled them to advance their rights within the framework of Israeli democracy. The second wave of Israeli feminism began in the 1970s, but feminist achievements became more significant only in the 1990s. Nonetheless, the descriptive level of representation of women in Israel is today low compared to the global average, both on the local and on the national level, in addition to establishment and non-establishment political processes, such as the struggle for religious-spiritual feminism (Herzog, 2006, 2011).

## Religious Feminism

Various scholars have linked the unequal character of society to written religious traditions (Stanton, 2002; see in detail: Yanay-Ventura, 2011, 2–3). It has been argued that Christianity, Judaism, and Islam ascribe women and men different statuses, manifested in different duties, privileges and roles, as well as different property ownership rights (El-Or, 2000). The conflict between feminism and religion is thus rooted in the model that religion created as its source of inspiration. As a result, radical feminism has asserted that freedom from patriarchy (also) requires abandoning religion, which was initially created in a patriarchal context (Hartman-Halbertal, 2000; Cicurel and Sharaby, 2007; Sharaby, 2016, 2017).

Yanay-Ventura’s (2014, 2016) analysis indicates that the discussion of religious feminism begins with acknowledging the existence of this conflict (Cohen, 2006). Several approaches that try to bridge feminism and religion have developed in the theoretical literature: adjusting feminism to religion by re-interpreting the concept of feminism; adjusting religion to feminism by redefining the concept of religion; and separating between religion and feminism (Yadgar, 2006; Ali et al., 2008). Similar representations are found in studies on religious feminist practices. The literature presents three strategies commonly employed by women in their religious feminist practices: a. women who respond to religious practices and are empowered by them; b. women who reject religious practices; c. women who respond partially, i.e., for strategic reasons respond to religion in some areas and to feminism in others (Yadgar, 2006).

Over the generations, Jewish communities throughout the world defined the ideal behavior rules for men and women (Libel-Hess, 2004, 14–15). Halakha sages often used the phrase “All glorious is the king’s daughter within the palace” (Psalms 45:14) to emphasize that women’s legitimate sphere of activity is within their homes. Since men were perceived as responsible for studying the Torah and performing rituals and customs that reinforced the connection to God, the appropriate sphere for their activity was the public Jewish sphere, which included the synagogue and the place of Torah study (Grossman and Haut, 1992, xxii). The Mishnah scholars exempted women from performing time-related commandments so that they could meet their family obligations (Ramon, 2001). Furthermore, the sages of the Mishnah and the Talmud did not view women as independent legal entities, and in many cases compared their legal status to that of minors (Hyman, 1976, 106).

Mishnah and Talmud sages forbade women to perform ritualistic practices such as being counted for a *minyan* (a quorum of ten men required for public prayer service), or being called up to the reading of the Torah in synagogue, for a number of reasons: a. their perceptions of female sexuality and the danger it poses for men; b. “A woman’s voice is a sexual incitement” (Berakhot 24: 71; Friedman, 2009), implying that a woman’s singing could provoke a man to have intercourse with a woman other than his wife; c. the impurity of menstruation, which was perceived as a threat to the social order (Douglas, 1966, 103; Don-Yehiya, 1997).

Orthodox feminism in Israel is related to the desire for enhanced involvement in religious life and practices (El-Or, 1998; Yanay-Ventura, 2016). Later, women’s prayer groups were formed, in which women read from the Torah scroll (Friedman, 2009). Another feature of Jewish orthodox feminism was renewal of rituals from which women were previously “exempted,” such as reciting Kiddush or Kadish (Vigoda, 2001). Rabbinical leadership by women is a phenomenon that has been developing in Israel’s modern orthodoxy over the past two decades (Yanay-Ventura, 2014; Libel-Hass, 2016). Since 1997, women have been ordained as rabbinical leaders in rabbinical courts (Gordin, 2005), and as “Halacha consultants” (Ganzel, 2005). Another model of female leadership is joint leadership by the community rabbi and his wife. Women also serve as heads of all-female colleges (Cohen, 2006).

## Women of the Wall

The Western Wall (Kotel) is the only wall that has survived from the four supporting walls that surrounded the old Temple in Jerusalem thousands of years ago. In Jewish tradition, the Western Wall is a spiritual and sacred area. It is located near a plaza (Plaza Wall) extending to the Western Wall itself. Prayers are held during the day, Rosh Chodesh (the Jewish New Moon), holidays and Bar Mitzvah ceremonies (a Jewish coming-of-age ritual). People also place small pieces of paper with prayers in crannies in the wall.

Religious prayers and rituals are conducted according to Jewish laws, as practiced by the Orthodox stream. Therefore, a fence divides between the men’s area and the women’s area (women’s assistance) and it is forbidden to cross the fence. The women are thus unable to worship in the same manner as the men. In recent decades, the Western Wall has been the focus of the struggle of the Women of the Wall, a group of women who are struggling to achieve religious gender equality in the Western Wall area and to practice their rituals in the women’s section similarly to the men. This is the origin for the organization’s name, the Women of the Wall.

The Women of the Wall is a feminist organization whose stated purpose is to allow women’s freedom of prayer at the Western Wall in the women’s section. The organization was established in 1988 when a group of women tried to hold a Rosh Hashana (Jewish New Year) prayer in the women’s section of the Western Wall, wrapped in a prayer shawl, putting on tefillin, dancing, carrying an Old Testament scroll (Torah) and reading it. These feminists’ rituals do not follow the patriarchal religious customs of the Orthodox and Ultraorthodox Jewish streams and upset some members of the Orthodox Jewish community, although they are regarded as legitimate by liberal and feminist movements who advocate for gender equality (Jobani and Perez, 2014; Reiter, 2017). Rosh Chodesh ceremonies have been being held in the women’s section of the Western Wall by the Women of the Wall since the establishment of their movement in 1988. A significant portion of the Orthodox public, and especially the Ultraorthodox Jews, are vehemently opposed to these ritual. The liberal movements, i.e., the Conservative and the Reform Jews in Israel and around the globe advocate and support the struggle (Libel-Hass, 2016). The body of knowledge on religious conflicts and their implications for the lives of women in general, and Jewish women in particular, is constantly growing (Lahav, 2015; Schnabel, 2017; Schnabel et al., 2018; Keysar and DellaPergola, 2019).

The current study follows critical studies which indicate that the real challenge in conceptualizing conflict lies in analyzing these phenomena, while exposing power relations within the patriarchal structure (Enloe, 2000; Charmé, 2005; Ackerly and True, 2010). Moreover, critical and feminist security studies indicate that conflict cannot be fully comprehended unless they are studied through the prism of how people experience them in a myriad way.

During the last three decades, the Western Wall was the focus of a struggle over its character on two levels: on the social level, in its religious and gender aspects, represented by the Women of the Wall (WOW) who advocated religious and gender pluralism; and on the political level – the struggle of the Reform and Conservative movements for equal footing at the Wall (Chesler and Haut, 2003; Libel-Hass, 2016; Reiter, 2016).

The first studies on the Women of the Wall were inside analyses by researchers who were among the first founders of this organization, i.e., Haberman (1997, 2012) who also discussed the feminist aspect of the religious realm in the Israeli context and Chidester and Linenthal (1995) who adopted Michel Foucault’s Theory of Power to convey the unavoidable competition or struggle over ownership, legitimacy and sacred symbols in such places. The WOW phenomenon raised the debate about the use of a divider (*mechitsa*) for women’s prayer at the Wall prior to 1948 (Shakdiel, 2002; Charmé, 2005; Shiloh, 2010). Shakdiel (2002), who is also a women’s rights activist but from a different school of thought, rightly portrayed the WOW as an undermining factor in Judaism and in “Israeli Ethnocracy.” Jobani and Perez (2014, 2017) studies pertain to the WOW’s impact on Israeli public discourse concerning the role of religion in Israel as a Jewish and democratic state. They suggest privatizing the administration of holy places for the different stakeholders and letting the State withdraw from religion.

We aim to expand the knowledge on women who fight for social and legal recognition of their rights as women, in religious conflict, i.e., to learn about their conflicts and processes during their activities and after facing highly difficult and unusual situations of violence and hostility. Therefore, the purpose of this study was to find the various voices of the WOW, and how Women of the Wall make sense of their experience in the complex conflict: What patterns can we trace regarding their attitude toward State policy makers and formal State authorities? What identity struggles, status conflicts, political and ethical dilemmas are prevalent in the WOW experiences as a consequence of their ritual and ceremonial activities? In this study, we explored the women’s voices and shed light on the complexities of the participants, woman who fight for women’s rights and religious freedom in a patriarchal environment and patriarchal norms. Our proposed methodology may extend debates in contemporary religious studies and political science by emphasizing the ambivalence and challenges faced by women when fighting for their religious, public, social, political, and personal rights.

## Methodology

### The Listening Guide Analysis

Feminist security studies, and particularly conflict studies, are intellectual approaches that highlight the voices of the marginalized and their often-silenced insecurities, as well as offering alternative narratives through feminist analyses. The methodology selected for the present study is rooted in these epistemological perspectives. The Listening Guide (Gilligan, 2015; Gilligan and Eddy, 2017) is a method of psychological analysis grounded in women’s experiences and involving multi-disciplinary methods.

The Listening Guide provides a multi-layered way of tapping into methodological, theoretical, epistemological, and ontological dimensions of the narrated subject (Harel-Shalev and Daphna-Tekoah, 2016). As a voice-centered, relational research approach, it aims to pay close attention to the subtleties of women’s voices and stories and access meaning in how individuals relate to themselves and to others. The Listening Guide methodology is distinctly different from traditional methods of coding in that one listens to the text of the interview, rather than categorizing or quantifying it (Tolman, 2001).

The analysis protocol includes four stages and is presented in detail in the section “Research Design and Methods” (Gilligan, 2015; Gilligan and Eddy, 2017). The collection of different voices that compose the voice of any given person – its range, harmonies and dissonances, distinctive tonality, key signatures, pitches, and rhythm – is embodied in culture and in relationships with oneself and with others. The Listening Guide was first applied in political science in studies focusing on women combatants’ experiences and in conflict studies (Daphna-Tekoah and Harel-Shalev, 2014; Harel-Shalev and Daphna-Tekoah, 2015).

### Research Design and Methods

The study conforms to internationally accepted ethical guidelines and relevant professional ethical guidelines and was approved by the head of the Ethics Committee of Ashkelon Academic College. After obtaining approval from the Ethics Committee (Ashkelon Academic College), In-depth, semi structured interviews lasting 1–2 h were conducted with each participant. To assure confidentiality, each participant was identified by a pseudonym.

We used snowball sampling for obtaining data through the observation and study of a focus group of 10 WOW leaders and through a series of personal interviews conducted with 16 women who participated in spiritual rituals, between 2018 and 2019. Both interviews and focus group dialogs were recorded and transcribed. All study participants were women, and all were Hebrew speakers. Some of them were both English and Hebrew speakers. The participants, who reside in various parts of Israel (both central and peripheral, and urban centers and small rural localities), were either native-born Israelis or immigrants, representing a broad variety of ethnic origins (North American, Middle Eastern, or African), religious views and socioeconomic backgrounds.

After obtaining the participants’ consent, the interviews were recorded and transcribed. All participants were assured of confidentiality, and names were changed to ensure anonymity. The semi-structured interview was initiated with the question: “What is the first association that arises in your mind when you think about the WOW?” Other questions followed regarding the participants’ experiences in the spiritual rituals.

### The Analysis Protocol

According to the Listening Guide methodology, the first phase is “listening to the plot.” In this phase, attention is paid to the entire story told by the interviewees. In the first step, one attends to what is happening: What stories are being told? Are there any repeated images, metaphors, or dominant themes? Any contradictions or absences? One also notes the larger social context within which the researcher and participant come together.

The narratives of the WOW women covered a description of joy from the spiritual ceremonies, the pleasure of feelings of sisterhood with their women partners. The women also shared their struggles for an equal right to a sacred space with us, in addition to rage and agony toward the opponents. The women described the Western Wall as a battle field. Moreover, the WOW narratives reflected their direct exposure to traumatic events due to their spiritual faith.

The researchers then document their reflexive elements, emotional and intellectual responses, thoughts and feelings to better understand how their responses to the interviewees might affect their perception and the analysis (Daphna-Tekoah and Harel-Shalev, 2017). During the analysis of the first listening, we researchers felt empathy and identification with the women who have been consistently attacked for the last 30 years due to their vision and beliefs.

The second phase of the Listening Guide involves composing the “I Poem,” which is a core feature of the approach that serves to identify the active self. The “I Poem” is obtained by cutting all phrases containing “I” from the transcripts and pasting them into a new text designated as the “I Poem” (Harel-Shalev and Daphna-Tekoah, 2016). The “I Poem” stage is devoted to tracing how the participants represent or speak of themselves during the interview. The researcher highlights each phrase in which the first-person form (I) was used in the interview transcript and then cuts and pastes the underlined passages into a new file to compose the interviewee’s “I Poem” (Gilligan, 2015).

The process can best be understood by presenting an example. For this, we present an examination of the interviews with Dina and Nira.

**I remember a specific traumatic moment**, when we once entered the Western Wall area, and the security guards said they had to search my body for a Torah Scroll. So, **I said to myself,** OK, because **I believe in upholding the law.** Then, when they started to search my body, and touched me all over, **I suddenly understood** what injustice is in the world! **I screamed** at the female guard “As a woman, think about what you are doing.” Then she called the male guard and he said to me “You are breaking the law.” **I clarified “If so, arrest me.”** This was my way of regaining control over my body and soul.

Listening to the rise and fall in Dina’s tone when she spoke, and reading Dina’s interview, highlights her use of the first-person form (I). Dina’s “I Poem” presents her self-presence during the traumatic moment of the interaction with the security guards, her ability to remember the traumatic act and her desire for justice.

Dina’s “I Poem” is given below:

I remember a specific traumatic moment,
I said to myself
I believe in upholding the law
I suddenly understood what injustice is in the world!
I screamed
I clarified “If so, arrest me.”

Nira shared her experience regarding the feeling of hostility from the ultraorthodox women during the ceremony with us, and her way of coping with the hostility.

My daughter was next to me, and **I was worried** for her so much…. **I felt stronger** when she stood near to me… **I knew** there would be female guards who will protect us and **I was looking** for them all the time… **I wanted** them to be there around us when **I entered the Kotel**. While praying **I looked at her**, **the [ultraorthodox] woman** and **I answered** her with praying. It was like praying dialogs. She prayed out loud in order to interfere with us, and **I responded to her with my own praying**… **I felt better**, it helped me.

Nira’s “I Poem:

I was worried
I felt stronger
I knew
I wanted
————
I entered the Kotel
I looked at her, the [ultraorthodox] woman
I answered her with praying
I responded to her with my own praying
I felt better”

The next phases of the Listening Guide analytic technique guide the interviewer(s) on how to identify the various voices of the interviewees. The interviewers trace how the participants present or speak of themselves during the interview and categorize the central voices used by the interviewees. The Listening Guide analysis of the focus group and the interviews identified two simultaneous interdependent voices: *The voice of trauma and insecurity*, and *the voice of sisterhood*. An expressive and loud voice was the voice of *fighting for women’s equal rights*. Examples of quotes from the transcripts that represent the various voices are presented below.

#### The Voice of Trauma and Insecurity

With reference to the rituals, the narratives of the WOW members reflected trauma due to the violence against them, especially by their opponents, both men and women, and was evidenced in the interviewees’ descriptions of their constant exposure to conflicts and violence.

##### Emily

The boys crowded closer and closer to us and then began running in front of us and stopping dead in their tracks – surrounding us, blocking us, preventing us from moving forward, trapping us on the stairs. In an instant I saw my limited options – there was no-one to help us. I had no idea how to call for police, the only possibility seemed to keep moving, to keep momentum going forward. So, I began to make double use of my heavy cane – lifting me from step to step, also using it to push the legs of these ultraorthodox boys out of my way. My arm was linked with Lea’s, pulling her forward as I went – I had the cane, she was “unarmed.” I was only peripherally aware of Lea’s male friend, who was trying to protect us, defend us, reason with the boys, shout at them to desist. I purposely didn’t look up at them – that would have slowed me down… I am clearly an older person, a person with some sort of handicap, a person who was having difficulty walking, and they were not showing deference, respect, care – far from it. Because we are the enemy. Were they caught up in a dilemma at all? Whether or not they felt any moral confusion or hesitation, they spent the better part of 15 min body blocking us, threatening – what? It’s taken me more than 2 weeks to put enough distance on this to realize that it was a traumatic experience.

##### Odelia

There are several levels – the violence and the injustice themselves, as well as the lack of attention and concern for violence and injustice. The moment when we lay down on the floor and hold the siddur [prayer book] are scalding. In those moments we experience it [the traumatic moment] and try to survive. We also have to prove that it is a problem [the violence], that it does happen, and it is not our fault. That is, to use these examples to prove over and over what we are going through. [To prove] to the government and to the media.

##### Varda

I joined the Women of the Wall following my daughter, who came home every time [after ceremonies] with experiences of coping with the violence. I felt it was wrong for me to be sending my daughter to a war zone when I was not there for her. I totally identify with the values we are fighting for.

##### Adina

Every time I entered the Western Wall area, I would be alert for the possibility that someone would hit me. Right in the square, outside the women’s section, and the men’s violence frightens me. But today I no longer have that, I’m not afraid. I got used to it [to the violence]. And the second thing is a prayer in which you stand, a complete prayer, and this is a mob of 13–14 years old girls [ultraorthodox] with no manners. I am already 60, almost a grandmother, how dare they? The values of Judaism, and the “respect the elders,” my hair is completely white, where are your values? So, they answered me – you’re Reform women…Your blood is permitted. I listened to the words and felt, in the air, that it will end with blood. With no blood it will not stop.

The ultraorthodox women internalize the patterns and values of the human (i.e., the male) society, that perceives women as an object. The challenge of feminism is against situations of self-alienation and constant consciousness contradictions in women’s experiences.

#### The “We Voice”: The Voice of Sisterhood and Solidarity

Interviewees used the “We Voice” to describe their sisterhood and feminism. The “We Voice” also represents their solidarity with liberal and secular values. The findings of our study should be understood in the context of the religious conflictual situation.

##### Sarah

I don’t give up easily (laughter), and I have been doing this for 8 years. Some have been better than others, but the determination to see it through and the determination not to let down the people who are depending on me, and not let the sisterhood down, because I think over all these years I developed a real sense of family and a real sense of love for my sisters.

##### Hellen

After Rosh Chodesh, I feel very strengthened, because I think we are making a very serious move, this is what I believe. I have friends, a group of women who support each other during prayers, each time we look at each other. I look at Talia and Talia says with her eyes, “I’m here for you.” Nataly with her apathetic eyes – do not worry, it’ll end. We are a strong group. The most difficult moment was when I saw that the other women had physical difficulties, when they [ultraorthodox women] attacked the elderly Women of the Wall. If they were attacking me, a person with physical power, it would be different. They attacked old women with no physical strength. It freaked me out.

We had the group, I caught a position at the end of the group and kept it so that they will not move me from there. I did not move…. Suddenly I found myself out of our group and understood that the orthodox women pushed me out of our ceremony. So, I could not pray with my group anymore, I was separated from them…. Although I fought against being moved, but they succeeded in breaking our human chain and I remained alone. Then I understood the degree of the violence.

##### Beth

I have a very clear duty to protect them all, because I have a responsibility for the lives and safety of all of them. I am responsible for the prayers so that they will be successful, all these responsibilities are on me. Everything else is marginal. When are there huge difficulties? When I cannot take responsibility, I cannot stand it. For example, when I see that something is happening to someone from our group and I cannot get to her quickly enough to help her. Or when I go to the police officer, and I tell him – “Some women are beating one of us, please, I want you to take her details so that I will be able to complain about her at the police station,” and the policeman replies “No”! I feel helplessness. I have nothing to do with it, and I do not know how to fight that feeling of helplessness. Everything that happens to us, usually, I try to find a solution for the problem, if not, in the next month, in the next prayer [I will find a solution].

#### Fighting for an Equal Spiritual Voice

I really believe we are on a spiritual mission. I know we have to do it, because I think the picture of a woman putting on tefillin and going up to the Torah is something that needs to be done.

Another voice was apparent in the women’s interviews, alongside expressing *the voice of trauma* resulting from exposure to the violence and hatred, *and the voice of sisterhood*. This is *the voice of fighting for women’s equal rights*. The women also expressed meaningfulness, passion and determination for their beliefs that women have a right to an equal spiritual voice.

This *voice of fighting for equality* was no less prevalent in their narratives and appeared alongside the other two voices. This voice exists in the complex historic connection between liberal democracy and feminism, as challenging liberal democracy and the exclusion mechanisms within it, and feeds on the human rights axiom that has accompanied such regimes from the beginnings of their existence. Concomitantly to its demand to implement this axiom on women, this voice also shaped and continues to shape the institution of liberal democratic citizenship and thus expanded and broadened the boundaries of democracy. The feminist struggle is not only against patriarchy, but also against deeply rooted perceptions among women who themselves internalize the viewpoints that exclude them from equal rights.

##### Tamar

I think that the religious feminist revolution in Israel is one of the revolutions that are still in their early stages. These are wonderful, fascinating revolutions, with very brave women. I believe that this revolution will lead to changes that we cannot imagine today, changes for the better. The masculine hegemony in religion must end. It must, it is not good for democracy, it is not good for the People of Israel, it is not good for women, it is not good for anyone except for the men themselves who are at the head of this hegemony and do everything, with lots of money, to preserve their advantage and their control. If there is something that needs to be done now to bring about a total change in Israeli politics, in the center of power of the ultraorthodox, in the field, in the exclusion, it is to empower this religious feminist revolution everywhere, in any manner, because this is what will break it.

“Fighting for equality! This is the real change that will bring about the Jewish subconscious – the one that women should bring about. Every time we are there and it’s hard for us, I do not hear the shouts or the disturbances, I really enjoy the prayer. I look at the women who shout and I pity them. I say – we’ll get to you and explain to you. I feel strong and I emerge strong, as long as I have hope, and we stay and do not give up, I feel victorious and this strengthens me.”

##### Ruth

I have to say that the situation helps me, even the violence, that makes it strong. We were in all kinds of periods with the Women of the Wall and that put us in a protective, strong, and just position. This position enables us to notice and see women. And that reinforces our ideology, and empowers our faith that what we are doing is right, and emphasizes the sense of injustice done to us.

##### Hava

Feeling our real mission helps me during violent situations. There is a stronger sense of mission than in non-violent situations. If I do not pray hard, and people will not hear my voice, there will be no prayers… The situation is historical and essential. It is amazing! And if there was no violence, I might not have been obligated to express my voice. I simply think that the presence of the Women of the Wall indicates the deep faith that women have equal rights. Not from a place of war, we do not come to fight that today it is this and tomorrow it is something else. It is not from a place of fighting. It is from a place of standing up for your faith and for this approach that women have equal rights and they deserve to pray and deserve to unite with the Torah and all the derived exaltation and that it can be reached in this scene, exactly as the men.

## Conclusion

*The police seemed to be skilled to frustrate my purpose. I could not strike them; my arms were being held. I could not even stamp on their toes, they seemed able to prevent that. Yet I must bring myself under arrest… A lecture on the law flashed through my mind. I could, even with all limbs helpless, commit a technical assault and so I found myself arrested and charged with spitting at a policeman. It was not a real spit but only, how shall we call it, a pout, a perfectly dry purse of the mouth. I could not have really have done it. Even to get the vote. Anyhow, there was no need, my technical assault was enough. She felt it a great comfort “to be written off as a spitfire”* (Pankhurst, 1905 in Atkinson, 2018).

The feminist revolution and the progress of women’s rights has changed dramatically in the last 150 years and affected women’s, men’s, and children’s lives around the globe. From the first feminists groups who fought for legal voting at the beginning of the 19th century, to the Women’s Liberation Movement, a radical multiracial feminist movement that grew directly out of the civil rights, antiwar, and related freedom movements of the 1960s. Its insight was that “the personal is political,” with the intention of decentralizing structure and men’s power, and its consciousness-raising method allowed it to grow rapidly and influence the feminist organizations and women’s struggles for civil rights (Evans, 2015).

The entry of women into the public sphere is evident today, and legal rights have become common practice. However, women and women’s rights regarding religion are still debated and females are still excluded from religious spheres. These spheres are usually controlled by hegemonic masculinity and by patriarchic norms and values (Bigelow, 2010). The significance of this study is vested in its contribution to a better understanding of religious feminism and the experience of women who seek religious equality in the religious domain, using the Women of the Wall as a case study. The case of these women enables us to explore nuances in their fight for equality, especially the fight to gain equality in a sacred, religious arena.

The article contributes to the feminist discourse about religious feminism and inclusion of women in the public domain. In this paper, we examined the distinction between the private and the public sphere in the context of power in a sacred space. While gender segregation in politics is ideological and has often been used to exclude and oppress women from power and politics positions (Herzog, 2006), we argue that religion should be viewed as a political-ideological viewpoint that serves as a primary cultural mechanism for excluding women from public action. Our study suggests reading the religious feminist struggle in a more nuanced expression of a dialectic process of creating a new identity and justice, which is not dichotomous, but rather is multidimensional and multifaceted.

The traumatic experiences that the WOW women were experienced during their fight for justice not only affected their political stance, but also their feelings of security and insecurity, both politically and personally. Listening to and analyzing the contrapuntal voices of women who fight for religious equality provide a way of systematically attending to the many voices embedded in their experiences (Daphna-Tekoah and Harel-Shalev, 2014; Gilligan, 2015).

Listening to the women’s voices and silenced voices using a feminist methodology enabled us to explore the feminist flame of these women and their persistent fight for the last 30 years against being second-class citizens in a sacred place. In May 2013, the Supreme Court of Israel ruled that Women of the Wall’s prayer gatherings at the Wall should not be deemed illegal. This fight for religious equality is similar to other battles of women who fought for feminist justice, such as the struggle for the right to vote by the suffragettes and the struggle of women against sexual abuse, rape and violence (Enloe, 2013).

Critical and feminist analyses have now brought to light important overlooked links between citizenship, rights and gender, with Feminist and critical studies aiming to reintroduce these silenced and marginalized voices. While women play crucial roles in cultural and political reproductions of national and other collectives (Yuval-Davis, 1997), the Listening Guide methodology provides a tool that can capture subconscious expression through investigation of voices that are usually not otherwise revealed within the context of the feminist discourse on Silences, Women’s Voices, and agency. This is particularly true for the feminist fight for religious justice, since it deepens the understanding of the nature of trauma among women who seek and fight for religious equality. As such, our study has political implications, since it has the potential to challenge dominant and repressive social practices. Similarly to the experiences of women in war zones, the WOW shared the violence they face with us, their personal distress and the traumatic moments of feeling danger and threat to their lives (Bowman, 2014; Daphna-Tekoah and Harel-Shalev, 2017). In conjunction with the voice of trauma and stress, we could trace the strength of the term sisterhood as an essential component in the WOW’s fight for equality. They also use the motto of “sisterhood is powerful” as an inspiring call to action and a practical application (Klein and Hawthorne, 1997).

The political and public voice of the women is increasing, and their voice is heard. However, the struggle of the Women of the Wall shows that these women in particular, and women in general, are still excluded from the religious space which is controlled by masculine hegemony. Similarly, to the struggles of the suffragettes for the right to vote, the case study of the Women of the Wall enables a broader understanding of the experiences of women struggling for their rights to expand their personal rights in the religious public space (Atran and Axelrod, 2008).

Listening to the voiced and silenced voices of the women enables learning about the struggle of these women, about their need to expand equality to other areas, such as the struggle of religious feminism and equality for women in religious rituals. Similarities arise between the challenges faced by the Women of the Wall and other feminist struggles for equality of women in religion in other places on the globe today (Bastin, 2002; Albera and Couroucli, 2012; Barkan and Barkey, 2014).

Since experiences and voices are signified through normative and ideological discourses (Harel-Shalev and Daphna-Tekoah, 2020), a sensitive voice-centered methodology such as the Listening Guide enable scholars to identify when and how the interviewees conform to – or resist – hegemonic discourses. Our empirical findings not only illustrate the utility of the Listening Guide technique in sacred places such as the Western Wall, but also challenge the conventional knowledge about women and religious feminism through interviewing women who fight for their justice.

The Listening Guide methodology provides a tool that can capture subconscious expressions through investigation of voices that are not usually otherwise revealed. We suggest that this methodology be integrated into the methods utilized in the Feminist arena and should be further explored in additional political contexts, such as security and insecurity in the public sphere.

## Data Availability Statement

The datasets generated for this study are available on request to the corresponding author.

## Ethics Statement

The study was reviewed and approved by the Ethics Committee of Ashkelon Academic College. This study was carried out in accordance with the recommendations of the Ethics Committee of Ashkelon Academic College, with informed consent from all participants. All subjects gave written informed consent. All names used in the manuscript are pseudonyms.

## Author Contributions

Both authors collected the data, analyzed, and wrote the manuscript.

## Conflict of Interest

The authors declare that the research was conducted in the absence of any commercial or financial relationships that could be construed as a potential conflict of interest.

## References

[B1] Ackerly B. True J. (2010). Back to the future: feminist theory, activism, and doing feminist research in an age of globalization. *Womens Stud. Int. Forum* 33 464–472. 10.1016/j.wsif.2010.06.004

[B2] Åhäll L. (2012). The writing of heroines: motherhood and female agency in political violence. *Secur. Dialogue* 43 287–303. 10.1177/0967010612450206

[B3] Albera D. Couroucli M. (2012). *Sharing Sacred Spaces in the Mediterranean: Christians, Muslims, and Jews at Shrines and Sanctuaries*. Bloomington: Indiana University Press.

[B4] Ali S. R. Mahmood A. Moel J. Hudson C. Leathers L. (2008). A qualitative investigation of muslim and cristian women’s views of religion and feminism in their lives. *Cultur. Divers. Ethnic Minor. Psychol.* 14 38–46. 10.1037/1099-9809.14.1.38 18229999

[B5] Atkinson D. (2018). *Rise Up Women!: The Remarkable Lives of the Suffragettes.* London: Bloomsbury Publishing.

[B6] Atran S. Axelrod R. (2008). Reframing sacred values. *Negotiat. J.* 24 221–246. 10.1111/j.1571-9979.2008.00182.x

[B7] Barkan E. Barkey K. (eds). (2014). *Choreographies of Sacred Sites: Religion and Conflict Resolution.* New York, NY: Columbia University Press.

[B8] Bastin R. (2002). *The Domain of Constant Excess: Plural Worship at the Munnesvaram Temples in Sri Lanka.* New York, NY: Berghahn Books.

[B9] Bigelow A. (2010). *Sharing the Sacred: Practicing Pluralism in Muslim North India*. New York, NY: Oxford University Press.

[B10] Bowman G. (2014). “Grounds for sharing - occasions for conflict: an inquiry into the social foundations of cohabitation and antagonism,” in *Shared Spaces and their Dissolution: Practices of Coexistence in the Post-Ottoman Sphere*, ed. Bryant R. (Oxford: Berghahn), 10–24

[B11] Bunch C. (2018). “Transforming human rights from a feminist perspective,” in *Women’s Rights, Human Rights*, eds Peters S. Wolper A. (New York, NY: Routledge).

[B12] Charmé S. L. (2005). The political transformation of gender traditions at the western wall in Jerusalem. *J. Fem. Stud. Relig.* 21 5–34. 10.1353/jfs.2005.0004

[B13] Chesler P. Haut R. (eds) (2003). *Women of the Wall: Claiming Sacred Ground at Judaism’s Holy Site.* Woodstock, VT: Jewish Lights Publishing.

[B14] Chidester D. Linenthal E. T. (eds). (1995). *American Sacred Space.* Bloomington, IN: Indiana University Press.

[B15] Cicurel I. Sharaby R. (2007). Women in the menstruation huts: variations in preserving purification customs among ethiopian immigrants. *J. Fem. Stud. Relig.* 23 69–84 10.2979/fsr.2007.23.2.69

[B16] Cohen T. (2006). Female jewish leadership: modern orthodoxy in israel as a test case. *Democratic Cult.* 10 251–295

[B17] Cook R. J. (ed.) (2012). *Human Rights of Women: National and International Perspectives.* Pennsylvania, PA: University of Pennsylvania Press.

[B18] Daphna-Tekoah S. Harel-Shalev A. (2014). “Living in a movie”—Israeli women combatants in conflict zones. *Womens Stud. Int. Forum* 44 26–34 10.1016/j.wsif.2014.03.002

[B19] Daphna-Tekoah S. Harel-Shalev A. (2017). The politics of trauma studies: what can we learn from women combatants’ experiences of traumatic events in conflict zones? *Polit. Psychol.* 38 943–957. 10.1111/pops.12373

[B20] De Beauvoir S. (2001). “*The Second Sex: Facts and Myths*,” Vol. 1 Hebrew trans. (Tel Aviv: Bavel and Yediot Aharonot). 10.1111/pops.12373

[B21] De Beauvoir S. (2007). “*The Second Sex: Woman’s Life Today*,” Vol. 2 Hebrew trans. (Tel Aviv: Bavel and Yediot Aharonot).

[B22] Don-Yehiya E. (1997). *The Politics of Accommodation: The Resolution of Religious Conflicts in Israel.* Jerusalem: The Floresheimer Institute.

[B23] Douglas M. (1966). *Purity and Danger: An Analysis of Concepts of Pollution and Taboo.* London: Routledge and Kegan Paul.

[B24] Ellison N. (1997). Towards a new social politics: citizenship and reflexivity in late modernity. *Sociology* 31 697–717. 10.1177/0038038597031004004

[B25] El-Or R. (2000). Blessed are you our lord, king of the universe, that didn’t make me a woman. *Ravgoni* 3: 30–35.

[B26] El-Or T. (1998). *Next Passover – Women and Scholarship in Religious Zionism.* Tel Aviv: Am Oved.

[B27] Enloe C. (2000). *Maneuvers: The international politics of militarizing women’s lives.* Berkeley, CA: University of California Press.

[B28] Enloe C. (2013). *Seriously!: Investigating crashes and crises as if women mattered.* Berkeley, CA: University of California Press.

[B29] Eschle C. (2001). *Global Democracy Social Movements and Feminist.* Boulder, CO: Westview Press.

[B30] Evans S. M. (2015). Women’s liberation: seeing the revolution clearly. *Fem. Stud.* 41 138–149.

[B31] Fogiel-Bijaoui S. (2011). “Introduction.” in *Democracy and Feminism*, ed. Fogiel-Bijaoui S. (Raanana: The Open University), 9–23.

[B32] Friedman H. (2009). “Teach me, my lord, to pray’: on the prayer group at neve daniel.” in *To be a Jewish Woman*, ed. Shiloh M. (Jerusalem: Urim), 241–335.

[B33] Ganzel T. (2005). (Not) Halachic adjudicators. *De’ot* 8: 15–17.

[B34] Gilligan C. (2015). The listening-guide method of psychological inquiry. *Qual. Psychol.* 2 69–77. 10.1037/qup0000023

[B35] Gilligan C. Eddy J. (2017). Listening as a path to psychological discovery: an introduction to the Listening Guide. *Perspect. Med. Educ.* 6 76–81. 10.1007/s40037-017-0335-3 28349266 PMC5383569

[B36] Goetz A. M. Jenkins R. (2018). Feminist activism and the politics of reform: when and why do states respond to demands for gender equality policies? *Dev. Change* 49 714–734. 10.1111/dech.12389

[B37] Gordin R. (2005). *The Cultural and Social Changes with Women’s Entry into the Field of Halachic Discourse*, Ph.D. dissertation, Bar Ilan University, Ramat Gan.

[B38] Grossman S. Haut R. (1992). “Preface”. in *Daughters of the King*, ed. Grossman S. Haut R.,. (Philadelphia, PA: The Jewish Publication), xxi–xxvii

[B39] Haberman B. D. (1997). Women beyond the wall: from text to praxis. *J. Fem. Stud. Relig.* 13 5–34.

[B40] Haberman B. D. (2012). *Israeli Feminism Liberating Judaism: Blood and Ink.* Maryland, MD: Lexington Books.

[B41] Handrahan L. (2004). Conflict Gender Ethnicity and Post-Conflict reconstruction. *Secur. Dialogue* 35 429–445.

[B42] Hansen L. (2006). *Security as Practice: Discourse Analysis and the Bosnian War.* London: Routledge.

[B43] Harel-Shalev A. (2017). Gendering ethnic conflicts: minority women in divided societies–the case of Muslim women in India. *Ethn. Racial Stud.* 40 2115–2134. 10.1080/01419870.2017.1277028

[B44] Harel-Shalev A. Daphna-Tekoah S. (2015). “Gendering conflict analysis: analysing Israeli female combatants’ experiences,” in *Female Combatants in Conflict and Peace*, ed. Shekhawat S. (London: Palgrave Macmillan), 69–83.

[B45] Harel-Shalev A. Daphna-Tekoah S. (2016). Bringing women’s voices back in: conducting narrative analysis in IR. *Int. Stud. Rev.* 18 171–194. 10.1093/isr/viv004

[B46] Harel-Shalev A. Daphna-Tekoah S. (2020). *Breaking the Binaries in Security Studies: A Gendered Analysis of Women in Combat*, Oxford: Oxford University Press.

[B47] Hartman-Halbertal T. (2000). Between tradition and revolution: women’s voices encounter jewish sources of authority. *Ravgoni* 3 44–49.

[B48] Herzog H. (2006). Religion and peacebuilding. *Sociol. Relig.* 67 331–333.

[B49] Herzog H. (2009). What is political? Feminist perspectives. *Theory Crit.* 34 155–163.

[B50] Herzog H. (2011). “NGOization of the Israeli feminist movement: depoliticizing or redefining political spaces?” in *The Contradictions of Israeli Citizenship*, eds Turner B. Ben-Porat G. (Abingdon: Routledge), 174–195.

[B51] Hill-Collins P. (2000). *Black Feminist Thought-Knowledge.* New York, NY: Routledge.

[B52] Hyman P. (1976). “The other half: women in the jewish tradition.” in *The Jewish Woman*, ed. Kaltun E. (New York, NY: Schocken), 105–113.

[B53] Jobani Y. Perez N. (2014). “Women of the wall: a normative analysis of the place of religion in the public sphere”. *Oxford J. Law Relig.* 3 484–505. 10.1093/ojlr/rwu021

[B54] Jobani Y. Perez N. (2017). *Women of the Wall: Navigating Religion in Sacred Sites.* New York, NY: Oxford University Press.

[B55] Keysar A. DellaPergola S. (2019). Demographic and religious dimensions of jewish identification in the us and israel: millennials in generational perspective. *J. Relig. Demogr.* 6 149–188. 10.1163/2589742x-00601004

[B56] Klein R. Hawthorne S. (1997). “Reclaiming sisterhood: radical feminism as an antidote to theoretical and embodied fragmentation of women,” in *Desperately Seeking Sisterhood: Still Challenging and Building*, eds Ang-Lygate M. Corrin C. Henry M. S. (New York, NY: Taylore and Francis), 57–70.

[B57] Kronsell A. (2012). Gender, sex and the postnational defense: *Militarism and peacekeeping.* Oxford: Oxford University Press.

[B58] Lahav P. (2015). The women of the wall: a metaphor for national and religious identity. *Isr. Stud. Rev.* 30 50–70.

[B59] Levi-Hazan Y. Harel-Shalev A. (2019). ‘Where am I in this story?’–listening to activist women writers. *J. Gend. Stud.* 28 387–401. 10.1080/09589236.2018.1485554

[B60] Libel-Hass E. (2016). *The Development of Liberal (Reform/Mitkademet and Conservative/Masorti) Judaism in Tel-Aviv: Organizational Patterns and Identities in the Congregations Beit Daniel and Tiferet Shalom (1991-2015).* Ramat Gan: Bar Ilan University.

[B61] Libel-Hess E. (2004). *Interpretations of ‘Tradition’: Building a Traditional/Conservative Community in Israel.* MA thesis, Ben Gurion University, Beer Sheva.

[B62] Love P. E. Smith J. Ackermann F. Irani Z. (2019). Making sense of rework and its unintended consequence in projects: the emergence of uncomfortable knowledge. *Int. J. Project Manag.* 37 501–516. 10.1016/j.ijproman.2019.02.004

[B63] Mott L. (2017). *Lucretia Mott Speaks: The Essential Speeches and Sermons.* Champaign, IL: University of Illinois Press.

[B64] Ramon E. (2001). “The first rib: women’s status in the mishnah as a basis for their status in the halacha,” in *The Third Rib*, eds Eran A. Ramon E. Shavit T. (Tel Aviv: Mofet Institute), 264–277.

[B65] Razavi S. (2000). *Women in Contemporary Democratization, Occasional Paper 4.* Geneva: United Nations Research.

[B66] Reiter Y. (2016). Feminists in the temple of orthodoxy: the struggle of the women of the wall to change the status-Quo. *Shofar Interdiscip. J. Jew. Stud.* 34 80–107.

[B67] Reiter Y. (2017). Contested holy places in israel-palestine: sharing and conflict resolution. *SAIS Rev.* 22 115–144.

[B68] Salvati M. Pistella J. Giacomantonio M. Baiocco R. (2018). Lesbians’ negative affect toward sexual minority people with stereotypical masculine and feminine characteristics. *Int. J. Sex. Health* 30 162–176. 10.1080/19317611.2018.1472705

[B69] Schnabel L. (2017). Gendered religiosity. *Rev. Relig. Res.* 59 547–556. 10.1007/s13644-017-0302-9

[B70] Schnabel L. Hackett C. McClendon D. (2018). Where men appear more religious than women: turning a gender lens on religion in Israel. *J. Sci. Study Relig.* 57 80–94. 10.1111/jssr.12498

[B71] Shakdiel L. (2002). “Women of the wall: radical feminism as an opportunity for a new discourse. *J. Isr. History* 21 126–163. 10.1080/13531040212331295892

[B72] Sharaby R. (2016). *The Renewal of a Tradition: The Seharane Celebrations in Israel.* Tel-Aviv: Resling Publishing.

[B73] Sharaby R. (2017). Significance of prenuptial rituals as ethnic definitional ceremonies among immigrants. *Adv. Anthropol.* 7 55–78. 10.4236/aa.2017.72005

[B74] Shepherd L. J. (2012). *Gender, Violence and Popular Culture: Telling Stories.* New York, NY: Routledge.

[B75] Shiloh M. (2010). “Feminist voices regarding gender equality in the struggle for the right to vote in the yishuv,” in *One Law for Man and Woman*, ed. Katvan E. (Ramat Gan: Bar Ilan University), 117–149.

[B76] Smith B. G. (ed.) (2000). *Global Feminism Since 1945.* New York, NY: Routledge.

[B77] Stanton E. C. (2002). *The Woman’s Bible: A Classic Feminist Perspective.* Mineola, NY: Dover.

[B78] Tolman R. M. (2001). “The validation of the psychological maltreatment of women inventory,” in *Psychological Abuse in Violent Domestic Relations*, eds O’Leary K. D. Maiuro R. D. (New York, NY: Springer Publishing Company), 47–59.

[B79] Vigoda A. (2001). “Simchat torah – a men’s holiday?” in *To be a Jewish Woman*, ed. Shiloh M. (Jerusalem: Urim), 198–201.

[B80] Yadgar Y. (2006). Gender, religion and feminism: the case of jewish israeli traditionalists. *J. Sci. Study Relig.* 43 353–370. 10.1111/j.1468-5906.2006.00311.x

[B81] Yanay-Ventura G. (2011). *Between Two Worlds to a New World: Orthodox Feminism in Israel.* Ph.D. dissertation, Bar Ilan University, Ramat Gan.

[B82] Yanay-Ventura G. (2014). Orthodox feminist identities in israel: a new look at feminism and a new look at orthodoxy. *Migdar* 3 1–24.

[B83] Yanay-Ventura G. (2016). Multifaceted religious feminism: the case of modern orthodox feminists in Israel. *Sociol. Anthropol.* 4 17–28. 10.13189/sa.2016.040104

[B84] Yuval-Davis N. (1997). *Gender and Nation.* London: Sage.

[B85] Zwingel S. (2016). *Translating International Women’s Rights.* Basingstoke: Palgrave Macmillan.

